# Cross-cultural adaptation and psychometric properties of the Thai version of the patient-reported outcomes measurement information system short form– depression 8a in individuals with chronic low back pain

**DOI:** 10.1186/s41687-024-00702-2

**Published:** 2024-03-04

**Authors:** Ruetaichanok Sansatan, Rotsalai Kanlayanaphotporn, Mark P. Jensen, Helena Correia, Prawit Janwantanakul

**Affiliations:** 1 Department of Physical Therapy, Faculty of Allied Health Sciences, Chulalongkorn University, Bangkok, Thailand; 2 Department of Rehabilitation Medicine, University of Washington School of Medicine, Harborview Medical Center, Seattle, Washington USA; 3 Department of Medical Social Sciences, Feinberg School of Medicine, Northwestern University, Chicago, Illinois USA

**Keywords:** Depression, Chronic low back pain, Cross-cultural adaptation, PROMIS, Psychometric properties

## Abstract

**Background:**

The study aimed to cross-culturally adapt the Patient-Reported Outcomes Measurement Information System Short Form v1.0 - Depression 8a (PROMIS SF v1.0 - Depression 8a) into Thai and evaluate its psychometric properties in individuals with chronic low back pain (CLBP).

**Methods:**

The PROMIS SF v1.0– Depression 8a was translated and cross-culturally adapted into Thai using the Functional Assessment of Chronic Illness Therapy translation methodology. Two hundred and sixty-nine individuals with CLBP completed the Thai version of PROMIS SF v1.0– Depression 8a (T-PROMIS-D-8a) scale and a set of measures assessing validity criterion domains. Structural validity, internal consistency, and test-retest reliability at a 7-day interval of the T-PROMIS-D-8a scale were computed and its construct validity was evaluated by computing correlations with the Thai version of Patient Health Questionnaire-9 (T-PHQ-9), Numeric Rating Scale of pain intensity (T-NRS), and Fear Avoidance Beliefs Questionnaire (T-FABQ).

**Results:**

Data from 269 participants were analyzed. Most participants were women (70%), and the sample had a mean age of 42.5 (SD 16.6) years. The findings supported the unidimensionality, internal consistency (Cronbach’s alpha = 0.94), and test-retest reliability (ICC _[2,1]_ = 0.86) of the T-PROMIS-D-8a. A floor effect was observed for 16% of the sample. Associations with the T-PHQ-9, T-NRS, and T-FABQ supported the construct validity of the T-PROMIS-D-8a.

**Conclusions:**

The T-PROMIS-D-8a was successfully translated and culturally adapted. The findings indicated that the scale is reliable and valid for assessing depression in Thai individuals with CLBP.

## Background

Chronic musculoskeletal pain is common and often disabling health condition, with a prevalence rate ranging from 11 to 36% [1, 2]. In Thailand, chronic low back pain (CLBP) affects about 27–30% of the population [3, 4]. CLBP is not only the leading cause of disability, but also reduces quality of life and work productivity, causing considerable economic burden to both individuals and society [5–7]. Previous research has indicated that individuals with CLBP had higher rates of emotional distress than those with acute low back pain and with general population [6, 8, 9].

Research supports a biopsychosocial model of chronic pain, which argues that pain and its impact are influenced by complex interactions between biological, psychological, and social factors [10]. Depression is one of the psychological factors that has been shown to play an important role in the progression and persistence of chronic pain [11, 12]. Approximately 20–25% of individuals with CLBP meet criteria for depression [9, 13, 14], and depression is significantly associated with higher pain severity, disability, worse recovery, and greater healthcare utilization in individuals with CLBP [15–17]. Previous research has also shown that early detection of depression and comprehensive treatments results in improved clinical outcomes [18, 19]. Therefore, depression should be routinely assessed in those with CLBP to inform treatment and maximize quality of life.

Commonly used measures of depression in research and clinical settings include the Beck Depression Inventory-II (BDI-II), the Center for Epidemiological Studies Depression Scale (CES-D), and the Patient Health Questionnaire (PHQ-9) [20]. These measures were developed using a classical test theory and contain items assessing the cognitive, affective, and somatic symptoms of depression (i.e., sleep, fatigue, and appetite) [21]. More recently, investigators have used Item Response Theory (IRT) to develop a number of patient-reported outcome domains, including depression [22, 23]. This effort has resulted in items that can be administered either via Computed Assisted Testing or as fixed-length static short forms. An 8-item short form (PROMIS SF v1.0 - Depression 8a) is commonly used. The eight items of this scale assess cognitive and affective depression symptoms, but exclude items assessed the somatic symptoms of depression, as these symptoms overlap with those associated with chronic pain. This makes the PROMIS SF v1.0– Depression 8a scale particularly useful for assessing depression in individuals with health conditions by reducing the influence of somatic symptoms on the final score [22, 24, 25]. The original English version of PROMIS SF v1.0 - Depression 8a shows strong psychometric properties, including reliability and validity [26–28].

The availability of translated versions of the PROMIS SF v1.0 - Depression 8a scale would be beneficial. The existence of multiple translations makes it possible to conduct cross-language, cross-country, and cross-cultural research on the role of depression in different health conditions, including chronic pain. Valid and reliable translations of the PROMIS SF v1.0 - Depression 8a would also allow for direct comparisons between individuals from different countries and who speak different languages with respect to the presence, severity, and correlates of depression across different cultures. Currently, there are several translated versions of PROMIS SF v1.0 - Depression 8a scale available. However, there is not yet a Thai language version of this scale. The purpose of the present study was to address the need for a Thai version of the PROMIS SF v1.0 - Depression 8a by translating the original version into Thai, and then evaluating its psychometric properties in individuals with CLBP.

## Methods

This study uses data from a survey which has also contributed data for another paper [29]. However, the other paper focused on presenting findings related to the translation and psychometric properties of other measures. This paper focuses on the translation and psychometric properties of the PROMIS SF v1.0 - Depression 8a scale. The current project was conducted in two phases. In the first phase, the English version of PROMIS SF v1.0 - Depression 8a was cross-culturally translated into Thai. In the second phase, we examined the psychometric properties of the resulting scale in a sample of individuals with CLBP. Ethical approval for the study was obtained from the Research Ethics Review Committee for Research Involving Human Research Participants, Health Sciences group, Chulalongkorn University (COA No. 097/65 and 208/65). Prior to data collection, each participant provided informed consent.

### Phase 1: Cross-cultural translation and adaptation

To develop a culturally appropriate translation, the Functional Assessment of Chronic Illness Therapy (FACIT) translation methodology was selected for this study [30]. The 11 FACIT steps are listed and described below:

#### Forward translation

The English version of the PROMIS SF v1.0 - Depression 8a was translated into the Thai version by two professional translators who are native Thai speakers. They were instructed to use appropriate and simple language appropriate for Thai culture.

#### Reconciliation

A third bilingual Thai translator reviewed the first two translations and suggested reconciliations of any differences found in those translations to produce a third translation. This translator documented the reasons for all decisions made.

#### Back-translation

A fourth translator who was a native English speaker and also fluent in Thai language performed a back-translation of the reconciled version into English. This translator did not have access to, nor did they have knowledge of, the original version, and was instructed to use simple language to capture the key meaning of the items.

#### Back-translation review

A native English speaker (MJ) who had experience with the English version of the PROMIS SF v1.0 - Depression 8a scale reviewed the back-translated instructions and items to evaluate how well these reflected the meaning of the original. The Translation Project Manager (PJ), who was a physical therapist and a native Thai speaker, provided comments on the differences between the back-translated version and the original version to ensure equivalent meaning.

#### Expert reviews

Three Thai health professionals in physical therapy who were native Thai speakers independently reviewed the results of each of the previous steps, and then selected the most appropriate translation for each item or provided alternative translations if needed.

#### Pre-finalization review

The Translation Project Manager (PJ) reviewed the results from each of the previous steps, identified problems and made comments on the step 5 translation to guide the Thai Language Coordinator (RK) to the next step.

#### Finalization

The Language Coordinator (RK), a physical therapist and a native Thai speaker, determined the final version by reviewing all the information in the previous translations and the comments made by Translation Project Manager (PJ) from the previous step. The Language Coordinator (RK) provided explanations for the choice of final translation as well as provided literal back-translation and polished back-translation for each item.

#### Harmonization and quality assurance

An English speaker (HC) who was involved in the development of the PROMIS Depression item bank and SF v1.0 - Depression 8a scale reviewed and evaluated the accuracy as well as the meaning of the final translation by comparing the final back-translations with the original version. She also confirmed that documentation of the translation process was complete.

#### Formatting, typesetting, and proofreading

Grammatical accuracy of the final translation of the PROMIS SF v1.0 - Depression 8a scale was independently checked by two proofreaders.

#### Cognitive testing and linguistic validation

The final Thai version of the scale was pretested with 10 individuals with CLBP to confirm that the meaning of each item was equivalent to the original version after translation. Each of the cognitive testing participants were asked to complete a questionnaire independently to provide feedback on the difficulty and appropriateness of each item. They were also asked to provide alternative wording for any items that they thought were difficult to understand.

#### Analysis of participant’s comments, and finalization of translation

The Translation Project Manager (PJ) collected and summarized the cognitive testing participants’ feedback. The Language Coordinator (RK) reviewed and proposed any final changes in the translation. Finally, the native English speaker who was involved in the development of the PROMIS Depression scale item bank (HC) conducted a final quality review, and the translation were finalized. This process resulted in the Thai version PROMIS SF v1.0 - Depression 8a (T-PROMIS-D-8a).

### Phase 2: Evaluation of reliability, construct validity, and structural validity of the T-PROMIS-D-8a

#### Participants and procedures

We recruited potential participants via referrals from physical therapists working in the outpatient physical therapy departments in hospitals and physical therapy clinics in Bangkok and nearby provinces from November 2022 to May 2023. Individuals were eligible if they were 18 years old or older, understood and communicated fluently in Thai, and had CLBP for at least 3 months that resulted in pain on at least half the days in the past 6 months. The low back area was defined as the area below the costal margin to the gluteal fold [31]. Those who presented a medical diagnosis from a physician indicating a history of severe lumbar spine pathology or serious medical conditions that could potentially impact their participation in the study were excluded. All potential participants who initially expressed an interest to participate in the study were screened by filling in a demographic questionnaire. Those eligible signed the informed consent form and administered paper-and-pencil versions of the T-PROMIS-D-8a, The Thai version Patient Health Questionnaire-9 (T-PHQ-9), Functional Rating Index (T-FRI), Numerical Rating Scale (T-NRS), and Fear Avoidance Beliefs Questionnaire (T-FABQ). They were given a stamped addressed envelope with the T-PROMIS-D-8a, and the Thai version of Global Perceived Effect (T-GPE) and were asked to complete the questionnaires after seven days and return them to the researcher. Because meaningful changes in depression, as measured by the T-PROMIS-D-8a, could occur within one week, only those who indicated little to no change in their condition (i.e., responded with − 1, 0, or 1 to the T-GPE) were used to evaluate test-retest reliability analyses.

#### Materials

The T-PROMIS-D-8a consists of 8 items assessing depression. The response options for each item range from 1 (“Never”) to 5 (“Always”). The responses to the items are summed into a total raw score, which is then transformed into a *T*-score using a conversion table with a mean of 50 and a standard deviation of 10 based on the original normative sample of the English version of the scale. *T*-scores ranging from 55 to 59, 60 to 69, and 70 or greater represent of mild, moderate, and severe level of depression, respectively (See https://www.healthmeasures.net).

The T-PHQ-9 assesses depression symptom severity. The item response options range from 0 (“Not at all”) to 3 (“Nearly every day”). Responses are summed into a total score that can range from 0 to 27. The scores ranging from 5 to 9, 10 to 14,15 to 19, and 20 or more indicate mild, moderate, moderately severe, and severe depression, respectively. It has been found to have acceptable psychometric properties in outpatients with the internal consistency (Cronbach’s alpha) of 0.79, indicating a good level of reliability [32].

The T-NRS is a commonly used measure of pain intensity. With this measure, participant rate their intensity of their pain on a 0 (“No pain”) to 10 (“representing an extreme level of pain”) scale. In the current study, participants were asked to rate their average pain in the past 7 days. Evidence supports the reliability and validity of the T-NRS [33].

The T-FABQ measures severity of fear-related beliefs. Item response options range from 0 (“Completely disagree”) to 6 (“Completely agree”). The total score ranges from 0 to 96. Responses to 7 and 4 items of 16 items are summed to create the T-FABQ Work scale and T-FABQ Physical Activity scale scores, which can range from 0 to 42 and 0 to 24, respectively [34]. The internal consistency (Cronbach’s alpha) of the T-FABQ total scores was 0.88, indicating a good level of reliability in Thai individuals with musculoskeletal pain [35].

The T-GPE asks respondents to indicate the amount of change in their condition, relative to a pre-specified time point. Response options can range from − 5 (“Vastly worse”) to 5 (“Completely recovered”) [36]. Evidence supports the reliability and validity of this scale in patients with musculoskeletal pain [36].

The T-FRI is a 10-item self-report scale assessing perceived disability in individuals with back and/or neck pain. The measure’s items assess pain intensity, pain frequency, and neck and/or back pain interference with daily activities. Item response options range from 0 (“No pain” or “Full ability to function”) to 4 (“Worst possible pain” or “Unable to perform this function at all”). The responses are summed, divided by 40, and the multiplied by 100 to get a total score that can range from 0 to 100; higher scores indicate more pain-related disability. The T-FRI has been shown to have good internal consistency (Cronbach’s alpha) of 0.86 in the current sample, indicating a good level of reliability [37].

#### Data analyses

Sample characteristics were described by computing frequencies and percentages (for categorical variables) or means and standard deviations (for continuous variables). We then determined the extent to which the T-PROMIS-D8a had problematic ceiling or floor effects (i.e., whether or not 15% or more participants had the lowest or highest possible scale scores [38].

Like all PROMIS measures, the PROMIS SF v1.0 - Depression 8a was developed specifically to meet unidimensionality assumption of Item response theory [24]. We employed confirmatory factor analysis (CFA) to evaluate the structural validity of the T-PROMIS-D8a, using the asymptotically distribution-free (ADF) method [39]. We planned to adjust the model as needed using modification indices by adding covariance between error terms if the modification indices exceeded 10 [40]. Model fit was evaluated using comparative fit index (CFI), Tucker-Lewis index (TLI), the root mean square error of approximation (RMSEA), and standard root of mean square residual (SRMR). Good fit model was obtained when CFI and TLI values > 0.95, RMSEA values < 0.06, and SRMR < 0.08 [41].

We evaluated the reliability of the T-PROMIS-D-8a using several approaches. First, we computed the internal consistency (Cronbach’s alpha) of the T-PROMIS-D-8a items. We determined a priori that a Cronbach’s alpha value of ≥ 0.70 would indicate acceptable internal consistency [42]. Second, we computed the measures test-retest reliability coefficient using the intraclass correlation coefficient (ICC _[2,1]_) between the initial and second assessments in those participants who reported no or very little change in depression from the first to second administration of the T-PROMIS-D-8a. An ICC_[2,1]_ between 0.75 and 0.90 is thought to indicate good reliability and an ICC_[2,1]_ ≥ 0.90 is thought to indicate excellent reliability [43]. The standard error of measurement for test-retest scale scores (SEM _test−retest_) was computed as $\sqrt {(\sigma _{{time}}^{2}+\sigma _{{residual}}^{2})}$ [44] and the minimal detectable change at 95% confidence (MDC_95%_) was calculated by MDC_95%_ = square root of 2 multiplied by SEM _test−retest_ x 1.96 [45]. Finally, the limit of agreement (LoA) was calculated as LoA = the mean change in scores between first and second administration ± 1.96 multiplied by standard deviation of these changes [46].

The construct validity of the T-PROMIS-D-8a was evaluated by computing Spearman’s rank correlation coefficients between the T-PROMIS-D-8a score and the three validity criteria measures. According to Cohen, correlation coefficients (*r*) of 0.10, 0.30, and 0.50 indicate small, medium, and large effect sizes, respectively. These values also correspond to weak, moderate, and strong associations between variables [47]. We hypothesized that if the T-PROMIS-D-8a had convergent validity, it should evidence strong positive association (i.e., *r*’s ≥ 0.60) with the T-PHQ-9. We also hypothesized that if the T-PROMIS-D-8a had discriminant validity it should demonstrate positive by only weak to moderate (i.e., *r*’s ≤ 0.40) with the T-NRS and T-FABQ [48].

All data analyses were performed using SPSS version 29.0 for Windows except for the CFA which was performed using AMOS version 29.0. A p-value < 0.05 was considered to be statistically significant.

## Results

### Cross-cultural translation and adaptation

The cross-cultural translation and adaptation process was successful in translating the instructions and all items of the PROMIS SF v1.0 - Depression 8a into Thai. These were found to be both understandable and appropriate for Thai culture.

### Demographic data and descriptive statistics

A total of 362 participants with CLBP were screened for eligibility and 354 expressed an initial interest in participation. However, 85 were not eligible and the other 8 ultimately declined participation. This left 269 participants for the Phase 2 analyses of data from the initial assessment. Demographic, pain history, and means and standard deviations of the study measures for this sample are presented in Table 1. All items of all questionnaires were completed with no missing data. The sample reported having moderate pain intensity on the T-NRS and moderate disability level on the T-FRI, on average. The average *T*-score of the T-PROMIS-D-8a was 53.8 (SD 9.0) indicating slightly higher depressive symptom severity than that reported by the normative sample, while the average the T-PHQ-9 scores was 8.4 (SD 5.2) indicating mild depressive symptoms, on average.

**Table 1:** Demographic, pain history, and study variable information for the study sample (*N* = 269)

Characteristic	n (%)	Mean (SD)
Self-identified sex		
Female	189 (70)	
Male	80 (30)	
Age (years)		42.5 (16.6)
Height (cm)		162.1 (8.9)
Weight (kg)		63.6 (14.2)
Body mass index (kg/m^2^)		24.1 (4.9)
Employment status*		
Working full time	202 (75)	
Unemployment	67 (25)	
Pain Duration (months)		33.7 (35.8)
Pain Intensity (T-NRS; 0–10)		
Current pain		5.9 (1.9)
Average pain (7-day)		5.9 (1.8)
Disability (T-FRI; 0-100)		45.5 (15.3)
T-PHQ-9 (0–27)		8.4 (5.2)
T-PROMIS-D-8a		53.8 (9.0)

*Percentages sum to > 100% due to rounding errors

*Note* T-NRS = Thai version of the Numeric Rating Scale; T-FRI = Thai version of the Functional Rating Index; T-PHQ-9 = Thai version of the Patient Health Questionnaire 9; T-PROMIS-D-8a = Thai version of the Patient-Reported Outcomes Measurement Information System Short Form - Depression 8a

### Structural validity

**Fig. 1:**
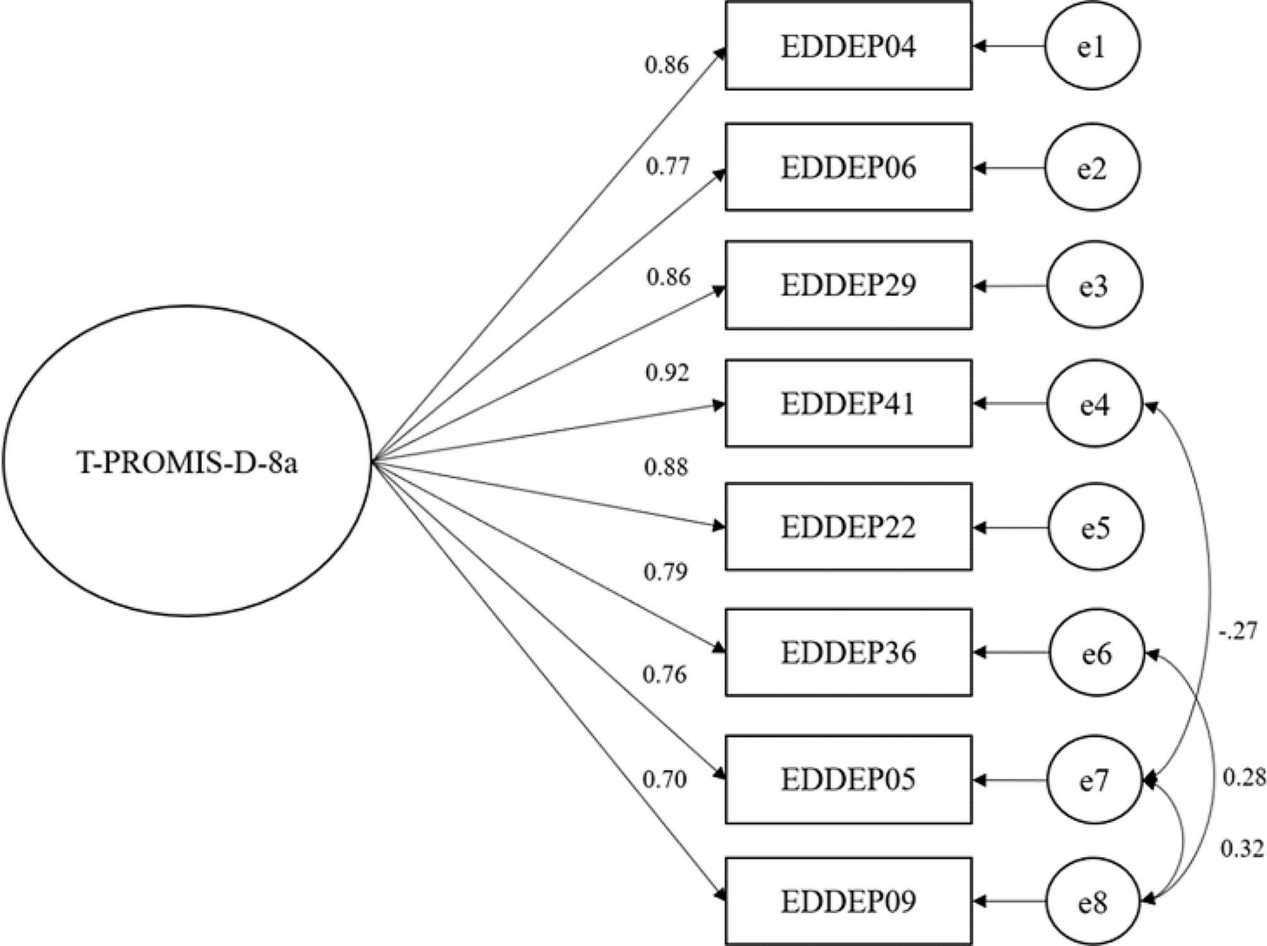
Single-factor model from the confirmatory factor analysis

The CFA using data from the 269 participants supported a single-factor model of the T-PROMIS-D-8a items. The initial model was adjusted and the modified model indicated good fit with all indices (CFI = 0.99, TLI = 0.98, RMSEA = 0.02, and SRMR = 0.02) Fig. 1 shows a single-factor model from the confirmatory factor analysis.

### Reliability, and ceiling and floor statistics

The Cronbach’s alpha of the T-PROMIS-D-8a in the current sample was 0.94, indicating an excellent level of internal consistency. 16% of the sample had the lowest possible score. For the second assessment, 101 participants reported that their pain condition over 1-week remained unchanged. The mean (SD) scores for T-PROMIS-D-8a at baseline and after a week were 53.7 (9.0) and 52.9 (10.2) respectively. Notably, the obtained ICC_[2,1]_ value of 0.86 indicated good test-retest reliability. The SEM_test−retest_, MDC_95%_, and LoA were 3.55, 9.85, − 0.83 ± 10, respectively.

### Construct validity

In support of convergent validity for the T-PROMIS-D-8a, it evidenced a strong association (*r* = 0.78) with the T-PHQ-9. In addition, the correlations between the T-PROMIS-D-8a and the measures used to evaluate discriminant validity criteria (i.e., the T-NRS and T-FABQ total score, activity subscale, and work subscales) were 0.21, 0.22, 0.07, and 0.23, respectively; all of these were indicated weak associations.

## Discussion

Using the FACIT methodology, which involves multiple steps designed to ensure that the translated questionnaire maintain and reflect the content of the original questionnaire accurately [30], we were able to translate the instructions and all eight PROMIS Depression SF v1.0– Depression scale items in a way that was both understandable to individuals from Thailand and appropriate for Thai culture. Consistent with the original English version of the PROMIS SF v1.0 - Depression 8a static scale, the findings confirmed the unidimensionality of T-PROMIS-D-8a and suggested that T-PROMIS-D-8a has acceptable reliability and construct validity when assessed in individuals with CLBP from Thailand.

The study results with respect to the unidimensional nature of the T-PROMIS-D-8a items are consistent with those using data from the original English version of the PROMIS SF v1.0 - Depression 8a [24] as well as other translated versions in different countries, including Norway, Korea, and China [49–51]. The T-PROMIS-D-8a was shown to have excellent internal consistency (Cronbach’s alpha: 0.94). This result is consistent with other studies in both clinical sample and general population (Cronbach’s alphas: 0.91-0.97) [27, 49–53].

The T-PROMIS-D-8a scale demonstrated potential problems with floor effects, with 16% of participants with the minimum possible score. A previous study on the original English version conducted in patients who underwent kidney transplant showed a higher floor effect of 21% which could be attributed to the fact that all participants were stable kidney transplants recipients [28]. Additionally, the Norwegian version demonstrated a higher floor effect of 30% among general population [50]. These findings suggest that the PROMIS SF v1.0 - Depression 8a is more suitable for use in patients with chronic clinical condition, rather than general population. However, the magnitude of floor effect with the T-PROMIS-D-8a is lower than the 50% value reported in a previous study conducting in Thai participants with CLBP using the 4-item of PROMIS Depression scale [54]. These findings suggest that while floor effects may be a concern in research using the T-PROMIS-D-8a, the T-PROMIS-D-8a still represents a marked improvement over the 4-items of PROMIS Depression scale with respect to this criterion. Therefore, whenever possible, the T-PROMIS-D-8a should be selected over the 4-item version for both research purposes and clinical practice.

The test-retest reliability of the T-PROMIS-D-8a was found to be good in the study sample. This finding is consistent with previous research using the original 8-item English version of the short form in patients with kidney transplants [28] and rheumatoid arthritis [27] who found equivalent results. When considered the findings indicating excellent internal consistency, the results indicate that the T-PROMIS-D-8a provides reliable results when administered over time. Relatedly, the findings regarding measurement error suggest that the difference between test-retest scores of the T-PROMIS-D-8a is expected to fall within the range of approximately − 10.59 to 8.93 T-score and a difference of at least 9.85 T-score points would be needed to determine that an observed change in depression severity is reliable. While, the 4-item of PROMIS Depression presented with a different of 13.75 T-score points [54]. This indicated that the T-PROMIS-D-8a is more sensitive in detecting meaningful changes.

In support of convergent validity, the T-PROMIS-D-8a evidenced a strong association with another measure of depression, the T-PHQ-9. The result indicated that T-PROMIS-D-8a and T-PHQ-9 were both designed to measure a similar construct. The finding is consistent with previous study in clinical sample (*r* = 0.83) [53]. However, the underlying concepts of these two measures are slightly different. T-PHQ-9 is composed of items that evaluate cognitive, affective, and somatic symptoms of depression, with a particular emphasis on somatic symptoms [55]. In contrast, T-PROMIS-D-8a primarily concentrates on assessing cognitive and affective symptoms, excluding somatic symptoms of depression. The approach aims to minimize the influence of somatic items that could potentially confound and inflate scores when evaluating individuals with chronic pain [24, 25]. As a result, the T-PROMIS-D-8a, which assesses only cognitive and affective symptoms of depression, may indicate a lower level of depression when evaluating individuals with chronic pain compared to the T-PHQ-9, which evaluates cognitive, affective, and somatic symptoms of depression. This notion might be supported by differences in levels of depression measured by these two measures in this study. At baseline, the mean T-PROMIS-D-8a scores indicated little or no depression. While T-PHQ-9 scores indicated mild depression in this current sample.

The study’s findings also supported the discriminant validity of the T-PROMIS-D-8a. Specifically, we found that the T-PROMIS-D-8a demonstrated positive but weak associations with valid measures of constructs that were not depression, including pain intensity and fear of pain. These results are consistent with prior research evaluating the discriminate validity of depression scales [56].

The current study has some limitations that should be considered when interpreting the results. First, this study only involved a convenience sample of individuals with chronic low back pain in two large public hospitals, one small public hospital, and five outpatient physical therapy clinics in the Bangkok metropolitan area and nearby provinces. The extent to which the findings generalize to individuals with CLBP from rural areas, or to individuals with other types of pain problems in Thailand are not known. Additional research that evaluates the psychometric properties of the T-PROMIS-D-8a in other populations is needed to determine the generalizability of the results. Second, the T-PROMIS-D-8a was not administered before and after a treatment known to impact depression. As a result, we are unable to evaluate the minimal clinically important difference for the T-PROMIS-D-8a. Further studies that address this issue would be beneficial. Third, we observed an unequal distribution of sex in the study, with 70% of the participants reporting that they were female. Different perceptions of depression between sexes might influence the structural validity of the model. Evaluating this possibility requires exploration in future studies.

## Conclusions

Despite the study’s limitations, the findings provide important new information regarding the reliability and validity of the T-PROMIS-D-8a. While more research to confirm the reliability of the results would be useful, the study findings indicate that the T-PROMIS-D-8a is a reliable and valid measure for evaluating depression in Thai individuals with CLBP. Additional research that replicates the current findings in samples of individuals with different chronic pain conditions as well as evaluates minimal clinically important difference of this measure in this current sample is needed.

## Data Availability

The manuscript does not contain any individual person’s data. The datasets used and/or analyzed during the current study are available from the corresponding author on reasonable request.

## Abbreviations

BDI-II: Beck Depression Inventory-II
CES-D: Center for epidemiologic studies depression scale
CFA: Confirmatory factor analysis
CFI: Comparative fit index
CLBP: Chronic low back pain
CTT: Classical test theory
FABQ: Fear Avoidance Beliefs Questionnaire
FACIT: Functional Assessment of Chronic Illness Therapy
FRI: Functional Rating Index
GPE: Global Perceived Effect scale
ICC: Intraclass correlation coefficient
IRT: Item Response Theory
MDC: Minimal detectable change
NRS: Numerical Rating Scale
PHQ-9: Patient Health Questionnaire-9
PROMIS: Patient-Reported Outcomes Measurement Information System
PROMIS SF: Patient-Reported Outcomes Measurement Information System Short Form
RMSEA: Root mean square error of approximation
SEM: Standard error of measurement
SRMR: Standard root of mean square residual
TLI: Tucker Lewis index
